# Therapeutic Implications of FABP4 in Cancer: An Emerging Target to Tackle Cancer

**DOI:** 10.3389/fphar.2022.948610

**Published:** 2022-07-11

**Authors:** Naihui Sun, Xing Zhao

**Affiliations:** ^1^ Department of Anesthesiology, The First Affiliated Hospital of China Medical University, Shenyang, China; ^2^ Department of Pediatrics, The First Affiliated Hospital of China Medical University, Shenyang, China

**Keywords:** metabolic reprogramming, fatty acid, tumor microenvironment, prognosis, cancer

## Abstract

Metabolic reprogramming is an emerging hallmark of tumor cells. In order to survive in nutrient-deprived environment, tumor cells rewire their metabolic phenotype to provide sufficient energy and build biomass to sustain their transformed state and promote malignant behaviors. Fatty acid uptake and trafficking is an essential part of lipid metabolism within tumor cells. Fatty acid-binding proteins (FABPs), which belongs to a family of intracellular lipid-binding protein, can bind hydrophobic ligands to regulate lipid trafficking and metabolism. In particular, adipocyte fatty acid binding protein (FABP4), one of the most abundant members, has been found to be upregulated in many malignant solid tumors, and correlated with poor prognosis. In multiple tumor types, FABP4 is critical for tumor proliferation, metastasis and drug resistance. More importantly, FABP4 is a crucial driver of malignancy not only by activating the oncogenic signaling pathways, but also rewiring the metabolic phenotypes of tumor cells to satisfy their enhanced energy demand for tumor development. Thus, FABP4 serves as a tumor-promoting molecule in most cancer types, and may be a promising therapeutic target for cancer treatment.

## Introduction

Metabolic reprogramming is the capability of tumor cells to reprogram their metabolic phenotype to satisfy the energy demands and activate metabolic signaling pathways for tumorigenesis. Tumorigenesis-related metabolic alterations encompass driving the metabolite influx to endow tumor cells increased capability to acquire the necessary nutrients and preferentially assigning the nutrients to metabolic pathways to support malignant behaviors. Tumor cells reprogram their metabolic phenotype including multiple aspects, including deregulated uptake of glucose and amino acids, utilization of glycolysis and tricarboxylic acid cycle intermediates for biosynthesis and NADPH production, altered regulation of metabolite-induced gene expression as well as complex metabolic interplay with the tumor microenvironment. Altered lipid metabolism is one of the most prominent characteristic changes in tumor metabolism. Lipids are a group of hydrophobic molecules that not only sustain energy and biomass production but also activate a series of oncogenic signaling pathways to participate in the regulation of tumorigenesis and progression (Currie et al., 2013). Alterations in fatty acid (FA) uptake, *de novo* synthesis, FA oxidation and storage as lipid droplets (LDs) are all considered to be critical for the survival, proliferation and metastasis of tumor cells (Snaebjornsson et al., 2020). Under metabolic stress, enzymes related to lipid metabolism are regulated due to alterations of metabolites and signaling pathways, making tumor cells increase lipids catabolic/anabolic activities, such as FA uptake, *de novo* synthesis, FA oxidation. FA synthesis and uptake supports tumorigenesis by providing necessary membrane phospholipids and signal molecules to sustain proliferation, and FA oxidation fuels tumor growth *via* production of ATP, NADPH and important intermediates. Tumor cells primarily obtain FAs through *de novo* FA synthesis or by exogenous uptake from the tumor microenvironment (TME) (Koundouros and Poulogiaanis, 2020). In terms of exogenous FA uptake, FAs require transporters to maintain solubility and efficiently mediate trafficking across the cellular membrane. Given that FA trafficking is a highly complex process and essential for many aspects of cellular function, these transporters for FA trafficking are implicated in the occurrence and development of cancer, which may open window for novel therapeutic strategies (Koundouros and Poulogiaanis, 2020).

Fatty acid-binding proteins (FABPs) belongs to a family of intracellular lipid-binding protein, binding hydrophobic ligands to regulate lipid trafficking, fluxes and metabolism (Storch and Corsico., 2008). FABP members are able to bind hydrophobic lipid ligands to function as cytoplasmic lipid chaperones to facilitate fatty acid solubilization, trafficking, and metabolism, interact with various membrane and intracellular proteins and regulate tissue and cellular specific lipid responses (Li et al., 2020). FABPs primarily participate in the regulation of uptake and trafficking of FAs from the adjacent stromal cells, regulation of specific metabolic signaling pathways and activation of metabolism-related gene expression (Furuhashi, 2019). More importantly, FABPs are essential for all aspects of FA metabolism, including promoting the trafficking of FAs for FA oxidation, regulating the transcriptional and enzymatic activity for FA *de novo* synthesis and LD storage. FABPs exhibit distinct patterns of tissue expression for the individual protein (Thumser et al., 2014). Given the importance of lipid metabolism involved in the tumorigenesis, FABPs are exploited for potential therapeutic targets in cancer management.

FABP4, also known as adipocyte fatty acid binding protein, is highly expressed in adipocytes, endothelial cells and immune cells (Furuhashi., 2019). During adipocyte differentiation, FABP4 interacts with hormone-sensitive lipase (HSL) or peroxisome PPAR-γ to regulate lipolysis, a process defined as the catabolism of triacylglycerols stored in cell lipid droplets (Garin-Shkolnik et al., 2014). FABP4 is also induced by lipopolysaccharide, advanced glycation end products and oxidized low-density lipoprotein during macrophage differentiation (Wang et al., 2011; Qiao et al., 2019). Exogenous FABP4 interacts with adipocytes to promote differentiation and facilitate p38/HSL-mediated lipolysis (Dou et al., 2020). FABP4 also mediates the maintenance and function of CD8^+^ tissue-resident memory cells. CD8^+^ tissue-resident memory (Trm) cells utilize exogenous FAs transported by FABP4 to sustain their oxidative metabolism for immunity (Pan et al., 2017).

**FIGURE 1:**
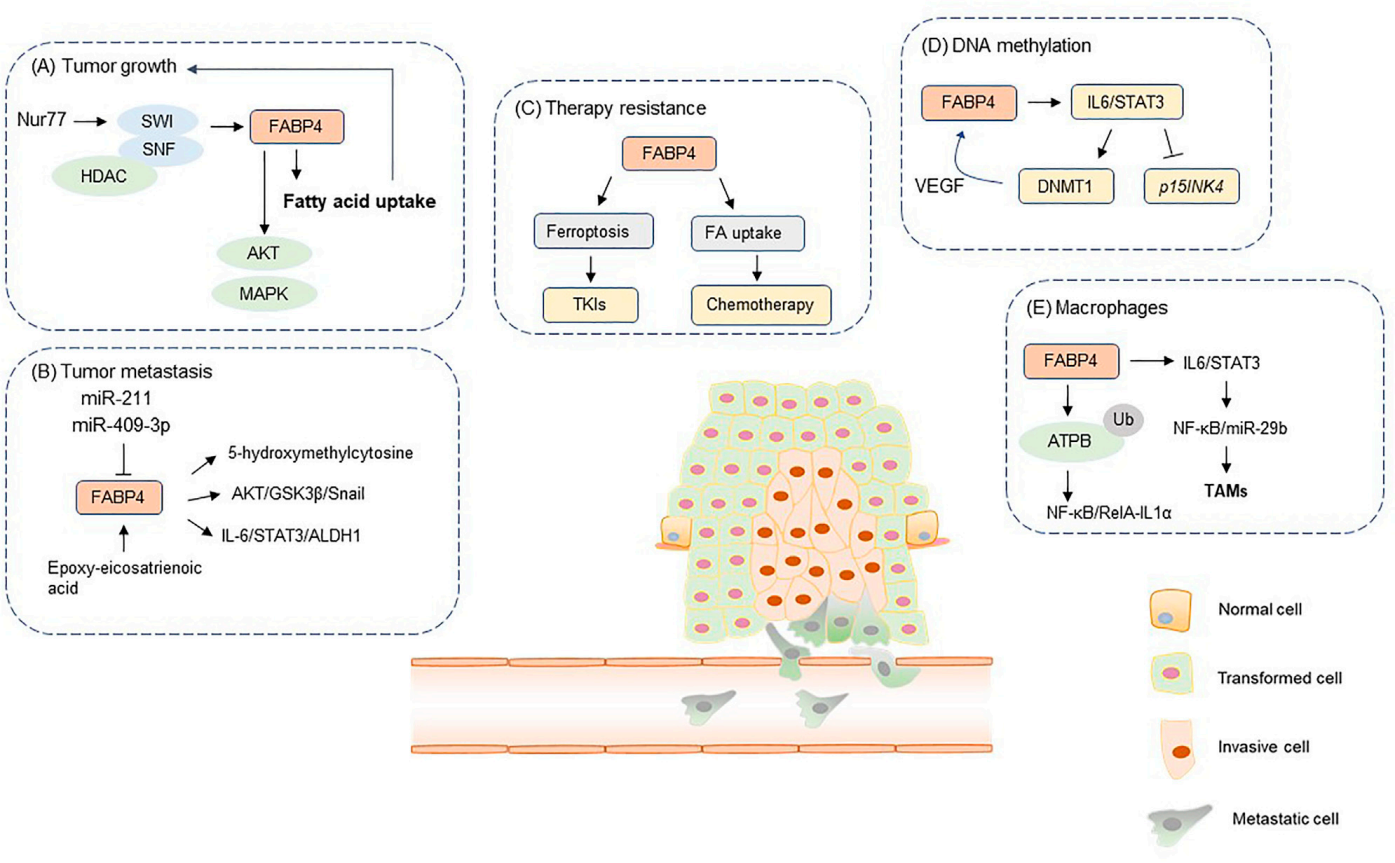
The role of FABP4 in tumor initiation and development. FA, fatty acid; TAMs, tumor-associated macrophages; TKIs, tyrosine kinase inhibitors. FABP4 participates in the regulation of diverse tumorigenic process, including **(A)** tumor growth, **(B)** tumor metastasis, **(C)** therapy resistance, **(D)** DNA methylation, **(E)** interaction between tumor cells and macrophages.

FABP4 plays an important role in the pathogenesis of a series of metabolic pathologies (Furuhashi, 2019). In particular, FABP4 levels are elevated in metabolic disorders, including obesity and metabolic syndrome (Furuhashi, 2019). Notably, circulating FABP4 level serves as a potential biomarker for these metabolic disorders (Furuhashi, 2019). In addition, aberrant expression of FABP4 has been observed in multiple cancer types (Guaita-Esteruelas et al., 2018). Importantly, FABP4 contributes to the tumor transformation, proliferation, metastasis and therapy resistance by enhancing lipid transport and activating diverse oncogenic signaling pathways (Guaita-Esteruelas et al., 2018). Moreover, FABP4 functions as a link between tumor cells and the components of TME, including adipocytes, macrophages and endothelial cells. In addition, the prognostic value and targeting strategies of FABP4 in diverse tumor types are also discussed in this review (Figure 1).

### Role of FABP4 in Tumorigenesis

#### FABP4 and Tumor Proliferation

Unlimited cellular proliferation is an essential hallmark of cancer (Hanahan D and Weinberg RA, 2011). In order to proliferate, tumor cells sustain increasing uptake and utilization of nutrients to support tumor growth (Hanahan D and Weinberg RA, 2011). Thus, exploring how metabolic phenotypes are intimately intertwined with rapid proliferation of tumor cells may provide novel therapeutic strategies for many types of aggressive tumors. FABP4 enhances tumor proliferation through multiple molecular mechanisms, including rewiring metabolic phenotypes, deregulating DNA methylation and upregulating oncogenic signaling pathways (Guaita-Esteruelas et al., 2017a; Yan et al., 2017; Yang et al., 2020).

FABP4 reprograms metabolic phenotypes to facilitate tumor growth. Orphan nuclear receptor Nur77 functions as a negative regulator for FA uptake and a tumor suppressor to inhibit tumor proliferation (To et al., 2012; Guan et al., 2020). Nur77 recruits SWI/SNF complex and HDAC1 to suppress the transcription of CD36 and FABP4, hampering breast cancer cells uptake of exogenous FAs and leading to the inhibition of cell proliferation (Yang et al., 2020). Proto-oncogene c-Src exerts tumor-promoting effects by activating oncogenic signaling pathways (Belli et al., 2020). Interestingly, FABP4 has shown inhibitory effects in Src-driven tumors. Src inhibition impairs LD formation by activating PPARγ-induced FABP4 expression and elevating reactive oxygen species (ROS) production, suggesting the critical role in Src/PPARγ/FABP4 axis in tumor proliferation (Hua et al., 2019).

DNA methylation is a common epigenetic modification that regulates gene expression, and aberrant DNA methylation patterns are considered a hallmark of cancer (Kulis and Esteller, 2010). Defects in DNA methylation lead to silencing of tumor suppressor genes and dysregulation of genes involved in DNA repair and chromosome stability, resulting in genome instability in cancers (Klutstein et al., 2016). Numerous studies have pointed out that obesity or high-fat diet regulates the methylome of cancer, which was associated biomarkers involved in DNA CpG methylation in tumors (Donovan et al., 2020). Dietary fatty acids alter the expression of DNA methylation modifiers, global CpG methylation, and gene-specific CpG methylation. For instance, supplementation of omega-3 polyunsaturated fatty acids could alter DNA methylation profiles by increasing methylation of IL-6 (Tremblay et al., 2017). FABP4, as a key mediator for fatty acid trafficking, has been found to be crucial for obesity-induced acute myelocytic leukemia (AML) growth through deregulated DNA methylation. FABP4 overexpression promotes IL-6 expression and STAT3 phosphorylation, resulting in DNA methyltransferase 1 (DNMT1) upregulation and loss of the *p15* ^I*NK4*^ tumor suppressor gene in AML cells (Yan et al., 2017). FABP4 inhibition by BMS309403 downregulated DNMT1 expression, impaired DNA methylation and rescued p15IN^K4B^ tumor suppressor gene by promoter DNA hypomethylation. Moreover, DNMT1 exerts positive regulatory effect on VEGF expression, and VEGF upregulation further increases FABP4 expression to form a vicious FABP4-DNMT1 loop to promote AML progression (Yan et al., 2017). Collectively, FABP4 coordinates fatty acid metabolism and DNA methylation to modulate the development of obesity-associated cancer.

FABP4 activates a series of oncogenic signaling pathways to enhance tumor proliferation. In breast cancer cells, exogenous FABP4 activates the AKT and MAPK signaling cascades, while inhibiting these pathways could impair FABP4-induced breast tumor growth (Guaita-Esteruelas et al., 2017b). Exogenous FABP4 can also activate PI3K/Akt pathway independent of fatty acid trafficking in prostate cancer to enhance tumor growth (Uehara et al., 2014). Collectively, these studies indicate a complex role of FABP4 in the activation of oncogenic signaling pathways.

### FABP4 and Tumor Metastasis

Tumor metastasis is responsible for most cancer-related deaths, and failure to limit the metastatic process is thus a huge obstacle for tumor elimination. Metastasis represents a complex multi-step process by which tumor cells disseminates from the primary tumor to a distant organ to establish the secondary metastases. FABP4 functions as a strong mediator in epithelial to mesenchymal transition, stemness, migration, invasion and metastasis (Gharpure et al., 2018; Zhao et al., 2019). The oncogenic functions of FABP4 are mediated by miRNAs in the process of regulating cell metastasis. Under hypoxia, miR-409-3p was downregulated, and FABP4 functions as a downstream target of miR-409-3p in ovarian cancer. FABP4 substantially increases the metastatic potential of ovarian cancer cells by altering the levels of 5-hydroxymethylcytosine in the DNA and the expression of key genes involved in the metastasis-related and metabolic pathways (Gharpure et al., 2018). In colorectal cancer, miR-211 inhibits cell migration, invasion, and EMT process by targeting FABP4 (Zhao et al., 2019). A novel FABP4/epoxy-eicosatrienoic acid (EET) dynamic that induces triple negative breast cancer (TNBC) metastasis has uncovered an opportunity for TNBC intervention. EET-associated nuclear translocation of FABP4 and nuclear accumulation of SREBP-2 or PPAR-γ significantly enhances TNBC cell migratory transformation and distal metastasis priming (Apaya et al., 2020). Therefore, targeting the EET-driven signaling pathways by galactolipid dLGG can significantly impair FABP4/EET-induced TNBC relapse and metastasis.

Epithelial-mesenchymal transition (EMT) and tumor stemness are major factors contributing to the metastasis of cancer cells. Elevated levels of circulating FABP4 in obese patients increase tumor stemness and aggressiveness. FABP4 upregulation leads to the activation of the IL-6–STAT3–ALDH1 signalling pathway and an increase in levels of STAT3-activating cytokines, which enhances the stemness of tumour (Hao et al., 2018). FABP4 also induces the EMT program in various cancer types. Exogenous FABP4 promotes EMT via the activation of AKT/GSK3β/Snail pathway in cervical squamous cell carcinoma, reorganizing the actin cytoskeletons in F-Actin staining and TGF-β induced EMT assays (Jin et al., 2018). CD36, a transmembrane glycoprotein, facilitates the trafficking of Fas (Pascual et al., 2017). CD36 can directly interact with FABP4 to modulate FA import and metabolism. The positive relationship between CD36 and FABP4 expression is critical for the rates of FA import to ensure that imported FAs can be efficiently transported to subcellular locations (Gyamfi et al., 2021). CD36 inhibition in ovarian cancer impairs adipocyte-driven FABP4 expression. However, FABP4 inhibition did not affect adipocyte-mediated CD36 expression, suggesting CD36 as an upstream regulator of FABP4 (Gyamfi et al., 2021). CD36 inhibition suspends adipocyte-driven EMT and stemness. These pro-metastatic effect of CD36 may correlates with FABP4.

### FABP4 and Therapy Resistance

Drug resistance is a major clinical issue that represents the principal cause of cancer-related deaths, with few targetable common pathways. The emergence of metabolic adaptation in resistance to therapeutics has paved the way to the exploration of targeting metabolic vulnerabilities of cancer cells to overcome drug resistance.

LDs have long been considered as inert depots for the storage of excess intracellular lipids. In cancer cells, LDs are often present in excessive amounts to satisfy the high demand of metabolic fuel and building blocks for membrane biosynthesis. LDs represent an underestimated organelle influencing intracellular pharmacokinetics and activity of anticancer tyrosine kinase inhibitors. For instance, LD enrichment of lung cancer cells by oleic acid supplementation potently reduces ponatinib activity, while LD depletion by the long-chain fatty acyl-CoA synthetase inhibitor triacsin C enhances the killing potential of this tyrosine kinase inhibitors (TKIs). Similarly, gefitinib-resistant NSCLC cells exhibit increased LD accumulation compared with gefitinib-sensitive NSCLC cells. Based on these findings, monitoring LD content within tumor cells may be essential for predicting therapy response to TKIs. Oxidative stress caused by hypoxia-reoxygenation exacerbated by TKIs leads to tumor reprogramming by increased lipid desaturation and transport. Ferroptosis is a new form of programmed cell death characterized by the accumulation of iron-dependent lipid peroxidation. FABP4 could enhance tumor transport for increased LD content, which coordinates with FA desaturation mediated by stearoyl-CoA desaturase-1 (SCD1), to protect tumor cells from oxidative stress-induced ferroptosis. This SCD1/FABP4 network provides tumor intrinsic antioxidant and anti-ferroptotic resources for survival and regrowth in a harsh TME (Luis et al., 2021).

Adipose tissues also play a critical role in the regulation of chemotherapeutic resistance. In the tumor microenvironment, adipose tissues survive under hypoxic and nutrient-deprived condition, which may lead to the alteration of cellular and molecular compositions. These alterations would inevitably affect chemotherapeutic responses of tumor cells growing in the neighborhood of tumor-associated adipose tissues. Adipocyte-derived conditioned medium has been found to reduce cytotoxic effects of chemotherapy to BC and PDAC cells. Besides, tumor stemness have been widely reported to be essential for mediating chemotherapy resistance and metabolic alterations in these cells might be the key to their ability to maintain stemness and drug resistance. Interestingly, adipocytes could upregulate expression levels of fatty acid transporter and fatty acid trafficking to induce tumor stemness. Co-culturing breast cancer cells with adipocytes significantly increase CD36 expression, CD36 activates STAT3 and ERK1/2 signaling pathways to induce the gene expression of EMT and stem cell traits. Recent studies have found that FABP4 specific inhibitor BMS309403 not only significantly reduces tumor burden in a syngeneic orthotopic mouse model but also increases the sensitivity of cancer cells toward carboplatin (Mukherjee et al., 2020). Taken together, targeting FABP4 may have a significant impact on the efficacy of chemotherapeutics through interactions with tumoral adipose tissues.

### Interaction of FABP4 With the Tumor Microenvironment

TME contains a group of diverse cell types, including immune cells, adipocytes, and endothelial cells, alongside cancer cells. Given that tumor cells constantly interact with surrounding cells, TME has been extensively reported in tumor occurrence and development. There is a growing number of studies supporting the potential of targeting the TME in cancer therapy.

### FABP4 and Adipocytes

In the TME, adipocytes have a complex interaction with tumor cells and therefore undergo a series of important phenotypic and molecular modifications to reprogram themselves to a state of cancer-associated adipocytes (CAAs) (Cao, 2019). Compared to normal adipocytes, CAAs display a series of characteristics, including fibroblast-like phenotypes, dispersed lipid droplets, releasing more leptin, adiponectin, interleukin-6, chemokine ligand two and chemokine ligand five and other adipokines (Zhao et al., 2020). FAs released from CAAs can be transported to tumor cells for energy production relying on FA oxidation and activation of downstream signaling pathways (Nieman et al., 2011). Therefore, CAAs contribute to tumor occurrence and development. Intracellular FABP4 is the one of the most critical players implicated in CAA-tumor cell interplay. This oncogenic effect is obvious in tumor cells within CAA-rich microenvironments including the breast, or tumors metastasizing to adipocyte-enriched sites such as gastric and ovarian cancers (Nieman et al., 2011; Tan et al., 2018). Adipocyte–cancer cell coculture mediates enhanced lipolysis in CAAs and FA oxidation in tumor cells, indicating CAAs as an energy source for the tumor cells (Nieman et al., 2011).

Some tumor cells including prostate and ovarian cancer preferentially home to and proliferate in the adipocyte-rich niche (Nieman et al., 2011). In these metastatic niches, FABP4 regulates the CAA-tumor interplay by mediating the transport of FAs between tumor cells and CAAs. The adipocyte-rich niches, such as bone metastatic niche or omentum, are metabolically active components that regulate the function of neighboring cells. For instance, once the prostate tumor cells reach the skeletal sites, lipolytic signals are activated in neighboring CAAs, leading to the release of lipids. The lipid transfer between marrow adipocytes and tumor cells can further fuel the growth and invasiveness of tumor cells at the metastatic sites by upregulating FABP4. Exposure of prostate tumor cells to adipocyte-derived factors has been proved to induce PPARγ-driven FABP4 expression (Uehara et al., 2014). The FABP4 upregulation can also be induced by cytokine IL-17A secreted by IL-17A-producing cells via p-STAT3 signaling in the metastatic process of ovarian cancer to omentum (Yu et al., 2020).

### FABP4 and Immune Cells

Tumor-associated macrophages (TAMs) represent one of the main tumor-infiltrating immune cell types and are generally categorized into either of two functionally contrasting subtypes, namely classical activated M1 macrophages and alternatively activated M2 macrophages. Macrophages infiltrate into solid tumor tissues and numerous evidences have pointed out that tumor-associated macrophages (TAMs) always acquire a polarized M2 phenotype to exert protumor functions, and the abundance of TAMs are correlated with poor disease prognosis (Li et al., 2019). Numerous evidences reveal that TAMs contribute to tumor progression by stimulating cell proliferation, metastasis and immune evasion (Ngambenjawong et al., 2017). Thus, eliminating TAMs may be a promising therapeutic strategy for cancer management. FABP4 was found to be significantly upregulated in a subset of TAM with a CD11b+F4/80+MHCII-Ly6C-phenotype. In these TAMs, FABP4 facilitates protumor IL6/STAT3 signaling through regulation of the NF-κB/miR-29b pathway (Hao et al., 2018). FABP4 increases FA transport and oxidation to elevate ROS generation and macrophage death (Zhang et al., 2017). In macrophages, FABP4 is critical for metabolism of excess FAs for ceramide synthesis. Ceramide is the precursor of all complex sphingolipids, which functions as a key regulator of cellular stress, senescence, and death. FABP4 inhibition in macrophage impairs FA-induced ceramide production, thus leading to suppressed cell death (Zhang et al., 2017). In neuroblastoma, FABP4 interacts with ATPB to facilitate ATPB ubiquitination in macrophages (Miao et al., 2021). The decreased ATP levels further deactivates NF-κB/RelA-IL1α pathway, contributing to the malignant phenotypes in neuroblastoma cells (Miao et al., 2021). Collectively, intracellular FABP4 is essential for the function of tumor-promoting macrophages.

CD8+Trm cells constitute the most abundant memory T cell subset. Precious studies have elucidated that the infiltration level of CD8+Trm cells are associated with antitumor immune responses, and the presence of CD8+Trm cells correlates with improved prognosis in patients with cancer. Importantly, CD8+Trm cells rely on exogenous fatty acid uptake and metabolism for cell survival. FABP4 and FABP5, the key transporters of fatty acids, have a critical role in the maintenance, longevity and function of CD8^+^ Trm cells to mediate protective immunity. However, gastric tumor cells outcompete Trm cells for lipid uptake and thus induce apoptosis of Trm cells, which could be reversed by blocking PD-L1 on cancer cells. Tumor cells highly express programmed death ligand 1 (PD-L1), which binds to receptor PD-1 expressed on activated T cells, thus leading to immune evasion. Anti-PD-1/PD-L1 therapy inhibits the binding of PD-1/PD-L1 to activate the exhausted T cells to enhance anti-tumor immunity. PD-L1 blockade decreases FABP4 and FABP5 expression in gastric tumor cells, while increases FABP4 and FABP5 expression in Trm cells, enhancing fatty acid uptake by Trm cells and sustaining survival of Trm cells (Lin et al., 2020).

### FABP4 and Angiogenesis

To increase the proliferative potential of cancer cells, complex vascular networks are required for tumor cells to sustain their malignant phenotypes (Viallard and Larrivee, 2017). Therefore, angiogenesis is a hallmark of malignant tumors and a promising therapeutic target for cancer treatment. Angiogenesis, a crucial step in tumor growth and metastasis, is regulated by various pro- or anti-angiogenic factors (De et al., 2017). Endothelial cell-FABP4, which is regulated by VEGF, mTORC1 and NOTCH1, exhibits a pro-angiogenic role by regulating expression of several key mediators of angiogenesis, including P38, eNOS, and stem cell factor/c-kit signaling to promote cell proliferation, survival, and migration, angiogenic sprouting (Elmasri et al., 2012; Harjes et al., 2017). In ovarian tumor xenografts, knockdown of endothelial FABP4 supports FA oxidation, elevates ROS and impairs angiogenesis (Harjes et al., 2017). In hepatocellular carcinoma, FABP4 promotes tumor development by upregulating the angiogenesis gene signature (Laouirem et al., 2019).

### Clinical Significance of FABP4 in Cancer

#### FABP4 as Prognostic Marker

Deregulated expression of FABP4 has been observed in multiple cancer types (Cui et al., 2019; Li et al., 2021). Dysregulated expression of FABP4 is associated with different clinic-parameters and prognosis, indicating FABP4 as a potential prognostic marker in different cancer types (Table 1). Upregulated intracellular expression of FABP4 is found in gastrointestinal stromal tumors, indicating a negative effect on the overall survival (OS) of these patients (Zang et al., 2021). Specifically, in cervical cancer, upregulated FABP4 expression in cancer tissues is associated with poor OS and pelvic lymph node metastasis, indicating the prognostic value of FABP4 in cervical cancer patients (Li et al., 2021). Among patients with non-small cell lung cancer, high expression of FABP4 is correlated with advanced tumor node metastasis stage (Tang et al., 2016). Although FABP4 has been found to be upregulated in most tumor types, FABP4 has also found to be downregulated in certain cancer. In liver cancer, FABP4 is downregulated than the adjacent normal tissue. Importantly, FABP4 was correlated with the tumor size, recurrence-free survival and OS (Zhong et al., 2018).

**TABLE 1:** Expression patterns and clinical significance of FABP4 in diverse tumor types.

Cancer Type	Expression of FABP4	Level	Clinical Significance	References
Bladder cancer	Downregulation	Protein	Lymph node metastasis	Mathis et al. (2018)
			Shorter OS	
Breast cancer	Upregulation	Protein	Tumor grade	Cui et al. (2019)
			Short DFS	
	Upregulation	mRNA	N/A	Hao et al. (2018)
Cervical cancer	Upregulation	Protein	Pelvic lymph node metastasis	Li et al. (2021)
			Poor OS	
Colon cancer	Upregulation	Protein	N/A	Tian et al. (2020)
Colorectal cancer	Upregulation	Protein	N/A	Zhang et al. (2019)
Glioblastoma	Upregulation	mRNA	N/A	Li et al. (2018)
Hepatocellular carcinoma	Downregulation	Protein	Short RFS and OS	Zhong et al. (2018)
Non-small cell lung cancer	Upregulation	Protein	Advanced TNM stage poor OS	Tang et al. (2016)
Pancreatic Ductal Adenocarcinomas	Upregulation	Protein	Lymph node metastasis	Luo et al. (2017)
			Poor prognosis	

Recent studies pointed out that circulating FABP4 levels have great clinical implications in cancer. Breast cancer patients display higher blood concentration of FABP4, implying elevated levels of circulating FABP4 as a specific promoter of the obesity-associated breast cancer (Guaita-Esteruelas et al., 2017a; Zeng et al., 2020). Another study also revealed that serum FABP4 levels are elevated in obese breast cancer patients than in non-obese breast cancer patients (Hancke et al., 2010). Moreover, in breast cancer, serum FABP4 is positively connected with tumor size and nodal-status (Hancke et al., 2010). Interestingly, circulating FABP4 may be persistently released into the circulation and reduce after the lesion is treated. Among patients with colorectal cancer, circulating FABP4 is higher than the levels in the control groups before surgery, and remarkedly reduces after operation (Zhang et al., 2019). Moreover, patients with elevated circulating FABP4 levels indicates higher risk of colorectal cancer. Collectively, circulating FABP4 opens new diagnosis and therapy perspectives for breast cancer, especially for obesity-associated breast cancer.

### Therapeutic Targeting of FABP4

FABP4 has emerged as a critical player in tumorigenesis, thus targeting FABP4 may provide a promising therapeutic target for cancer treatment. FABP4 has been targeted using various approaches, including small molecule inhibitors, siRNAs and short hairpin RNAs (Table 2). Currently, no clinical trials have underwent to evaluate the efficacy of FABP4 inhibition in the clinical setting. Thus, clinical trials should be accelerated to test the therapeutic effects of FABP4 inhibitors in diverse types of cancer, which may implement the options of therapeutic strategies for cancer patients in clinical practice. BMS309403, which was first identified a drug for metabolic syndrome, can effectively impair the growth and metastases of tumor in several mouse models by targeting both tumor and stromal cells (Mukherjee et al., 2020). Further studies are required to identify the responsive tumor types. In addition, combination of FABP4 inhibitors with anti-tumor drugs like chemotherapy and TKIs could augment the anti-tumor effect of traditional (Luis et al., 2021). More studies should be focused on the oncogenic role and underlying mechanisms of FABP4 in cancer development to improve the clinical outcomes of cancer patients. In particular, deep understanding of the potential adverse effect is required to ensure the safety of combination therapies.

**TABLE 2:** Therapeutic targeting of FABP4 evaluated in different cancer types.

Methods	Cancer Type	Results	References
Inhibitor	Breast cancer	Inhibiting growth and metastasis	Uehara et al. (2014)
BMS309403			
	Cholangiocarcinoma	Inhibiting metastasis	Yang et al. (2021)
	Colon cancer	Inhibiting metastasis	Tian et al. (2020)
	Hepatocellular carcinoma	Inhibiting growth and metastasis	Yan et al. (2018)
		Inhibiting growth and metastasis	Laouirem et al. (2019)
	Leukemia	Inhibiting growth	Yan et al. (2017)
	Liver cancer	Inhibiting growth and self-renewal	Wang et al. (2019)
	Ovarian cancer	Inhibiting growth and metastasis	Mukherjee et al. (2020)
		Increasing sensitivity to carboplatin	
	Prostate cancer	Inhibiting metastasis	Nie et al. (2017)
Inhibitor BD62694	Ovarian cancer	Inhibiting recurrence	Tian et al. (2020)
siRNA	Hepatocellular carcinoma	Inhibiting growth and metastasis	Wang et al. (2019)
	Leukemia	Inhibiting growth	Yan et al. (2017)
	Ovarian cancer	Inhibiting metastasis	Gharpure et al. (2018)
shRNA	Triple-negative breast cancer	Inhibiting metastasis	Apaya et al. (2020)

## Conclusion

Collectively, FABP4 has been found to be upregulated in most cancer types, and correlated with poor prognosis. FABP4 primarily functions as a promoter for tumor proliferation, metastasis and drug resistance. More importantly, FABP4 is a crucial driver of malignancy not only by activating the oncogenic signaling pathways, but also rewiring the metabolic phenotypes of tumor cells to satisfy the increased energy demand for tumor development. Thus, FABP4 serves as a tumor-promoting molecule in most cancer types, and may be a promising therapeutic target for cancer treatment.

## Abbreviations

acute, acute myelocytic leukemia; AML, cancer-associated adipocytes; CAAs, CD8^+^ tissue-resident memory cell; Trm, DNA methyltransferase 1; DNMT1, epithelial-mesenchymal transition; EMT, epoxy-eicosatrienoic acid; EET, fatty acid; FA, fatty acid-binding proteins; FABPs, hormone-sensitive lipase; HSL, lipid droplets; LDs, overall survival; OS, programmed death ligand 1; PD-L1, reactive oxygen species; ROS, stearoyl-CoA desaturase-1; SCD1, triple negative breast cancer; TNBC, tumor-associated macrophages; TAMs, tumor microenvironment; TME, tyrosine kinase inhibitors, TKIs.
